# Mathematical Identification of Influential Parameters on the Elastic Buckling of Variable Geometry Plate

**DOI:** 10.1155/2013/268673

**Published:** 2013-11-24

**Authors:** Mirko Djelosevic, Jovan Tepic, Ilija Tanackov, Milan Kostelac

**Affiliations:** ^1^Faculty of Mechanical and Civil Engineering Kraljevo, University of Kragujevac, 36000 Kraljevo, Serbia; ^2^Faculty of Technical Sciences Novi Sad, University of Novi Sad, 21000 Novi Sad, Serbia; ^3^Faculty of Mechanical Engineering and Naval Architecture, University of Zagreb, 10000 Zagreb, Croatia

## Abstract

The problem of elastic stability of plates with square, rectangular, and circular holes as well as slotted holes was discussed. The existence of the hole reduces the deformation energy of the plate and it affects the redistribution of stress flow in comparison to a uniform plate which causes a change of the external operation of compressive forces. The distribution of compressive force is defined as the approximate model of plane state of stress. The significant parameters of elastic stability compared to the uniform plate, including the dominant role of the shape, size, and orientation of the hole were identified. Comparative analysis of the shape of the hole was carried out on the data from the literature, which are based on different approaches and methods. Qualitative and quantitative accordance of the results has been found out and it verifies exposed methodology as applicable in the study of the phenomenon of elastic stability. Sensitivity factor is defined that is proportional to the reciprocal value of the buckling coefficient and it is a measure of sensitivity of plate to the existence of the hole. Mechanism of loss of stability is interpreted through the absorption of the external operation, induced by the shape of the hole.

## 1. Introduction

Thin-walled plates are the basic components of a large number of modern supporting structures of open and closed type or profiled and box girder that are used in the construction of bridges, airplanes, boats, and other authorized facilities. Plate shaped elements of no uniform geometry are often incorporated in these structures which implies a variation in thickness and existence of various shapes holes. Requirements for using the plates with perforated holes and stepwise variable thickness result from functional, assembly, service, control, and other conditions, and often they are the result of weight minimization in order to achieve economic effect in the process of optimum design of structures. Mathematical identification of phenomenon of plate stability and general stability of girder and columns as their components is based on the application of the methods of Kantorovich and Ritz. The first method is based on solving partial differential equations of the fourth order (1), and it is derived from the equilibrium conditions of thin plate of constant geometry. The second method is based on the principle of minimum total potential energy, which provides a stable state of plate balance. Theoretical basis for both methods were systematically presented in [1–3]. The issue of stability of thin-walled elements occupies an important place in the process of supporting structures design [4].

For plates of constant thickness affecting parameters include geometric sizes, supporting conditions, and load conditions [5–7], while the stability of the element with a hole in addition to these characteristics is affected by the shape, size, and position of the hole [8, 9].

Most researches in the area of stability refer to the idealized case of a simply supported plate. In contrast to this is the plate with restraint edges along which the displacements and rotations are disabled. These two extreme and diametrically opposite cases practically very rarely occur in pure form, but they are most often occur in combination with other basic supporting conditions. In the case of real girders there is a case of elastic supported plate meaning that along the individual edges are beams of certain bending and torsional rigidity. Analysis of constant geometry plate shows that the supporting conditions greatly affect the stability [7]. In addition, implementation of staying is the way to increase stability [10–13].

Researches of plates with holes and/or stepwise variable thickness as components of girder are of special interests [14–24]. It is important to emphasize that very important methods are those having universal application in the study of the stability of plates and shells. It refers to the elements of the support that may have more holes, usually arranged equidistant (perforation) as well as sudden or gradual changes in thickness.

Rational design of structures requires the use of these structures which includes the identification of parameters influencing stability. In the last ten years, development of computer techniques based on the use of finite elements method (FEM) has enabled researchers to make a significant contribution to this phenomenon [25]. Researches [18, 22] show that the existence of the hole leads to a reduction of buckling coefficient for a certain value, which depends only on the shape, size, and position of the hole. To this the fact that the distance between the holes in perforated plates is also a parameter which in interaction with others affects the value of the critical buckling stress should be added. Due to the complexity of the mathematical model between the buckling coefficients, that is, minimum value of the critical buckling stress and characteristic parameters of the hole, researchers have mostly kept on semianalytic, approximate, and numerical methods and expressions obtained experimentally.

## 2. Analysis and Problem Formulation

A significant number of researchers have examined the phenomenon of linear or elastic buckling of plates indicating the importance of such an analysis in the design of structures. Studies of plate elements of girder or columns with perforated holes are of particular interest. These studies are realized using the finite element method, finite strip method [26–28], and experimental tests. Due to the geometric complexity and stress complexity the study of such problems mathematically is aimed to the partial analysis of individual components.

Analysis of the elastic buckling of plates a number of researchers has relied on the application of von Karman's theory based on the fourth-order partial differential equation [1, 2, 29]:

(1) $$\begin{array}{c} \nabla^{4}w=-\frac{1}{D}\left(N_{x}\frac{\partial^{2}w}{\partial x^{2}}+N_{y}\frac{\partial^{2}w}{\partial y^{2}}+2N_{xy}\frac{\partial^{2}w}{\partial x\partial y}\right). \end{array}$$

The function of the deflection depends on the type of load and boundary conditions and it is presented as a linear combination of trigonometric and hyperbolic functions. As analyzed plates have ideally planar geometry without transversal pressure and initial curvature, integration constants that define the contour conditions remain indefinite. 

In this case, the deflection function with indefinite coefficients can be used to determine the coefficient of buckling. Under the influence of compression load plate is in the position of stable equilibrium until a critical intensity when it suddenly loses stability and fracture occurs. This situation is a consequence of the idealization of the initial state of plate geometry. The application of this method is limited to a plate of constant thickness.

Numerous studies of linear buckling plates, especially those that are based on mathematical models, refer only to the problem of uniform plates. Study subject are plates of constant or variable thickness in one or both directions under the influence of combined plate loads. Analytical solution for pressure plate of variable thickness composed of two segments of the same length is given in [5]. The energy method based on the Rayleigh-Ritz principle is applied to analyze the plate [29, 30]. More complex cases are where the plates of gradual variable thickness in two normal directions are considered by the finite difference method [6].

Thin-walled elements are potentially critical places of the structure due to the interactive effect between planar state of stress and elastic buckling phenomenon. The existence of holes in such elements followed by a stress concentration generally reduces their strength to buckling. Elastic buckling is the first step in the analysis of stability of structures. Measures taken to increase the critical stress of elastic buckling effect on favorable on behavior in the zone of inelastic buckling and on mechanism of boundary conditions.

The subject of this paper simply supported uniaxial loaded rectangular plate with square, rectangular, and circular holes as well as slotted holes. Elastic buckling of plates with so-defined geometry and load conditions is modeled in terms of energy method (detailed information is given in Section 3). The mathematical model is based on the principle of minimum total energy of plate that provides a state of stable equilibrium. Model of the elastic buckling of plate with a hole is tightly coupled with the model of a planar state of the considered element. The aim of the authors is to verify and complement the existing results regarding the elastic buckling of plates with various shapes holes. The analysis based on the physical and mathematical model enables exact identification of mechanism of elastic stability.

## 3. The Mathematical Model

The approach, in this paper, based on an analytical approach, requires forming a mathematical model according to defined physical conditions of supporting and load. Beside the model of elastic stability, it is necessary to form a model of plane state of stress of plate with rectangular hole. In this paper, the research is directed to analyze plates with holes of different shapes in the domain of elastic material behavior. Supporting structures are formed by different geometry plates interconnected into a functional entirely. 

The model formed on the basis of the plates system provides a real picture of the behavior of the whole cross section. Approach to solving was given in [31], where the problem of the static load capacity of box girder partially exposed to horizontal loads is analyzed. Partial analysis of stability of girder includes independent examination of certain plates [32], which greatly simplifies the mathematical model, which will be presented in this chapter.

### 3.1. Core of the Buckling Problem

The mathematical model is based on the application of energy method using total potential energy of plate. The most general application of the energy method refers to the case of transversely loaded plates and/or plates with initial curvature simultaneously exposed to the effects of plane forces. Transversal and plane forces represent known external load whose effect is manifested through external work *E*_*W*_, which leads to a deformation of plate. As a result of external load we have occurrence of deformation energy *E*_*I*_, manifested through the process of bending and torsion which leads to the induction of an additional plane forces due to the existence of connections of plate supports, if any. Transversal loading affects the bending of plate. Plane forces affect increase or reducing of the plate deflection in dependence on whether they are pressed or tightening. The existence of initial curvature is equivalent to the existence of a fictive transversal load [1].

The mathematical model is formed based on the following assumptions:

- considered plates have ideal geometry (without significant warping);
- behavior of isotropic plates is in the area of elastic stability;
- the effect of supporting to inducing forces in a plate's plane is ignored;
- the impact of the external transversal loading is eliminated.

Generally, the energy balance of plate, which is located in the state of stable equilibrium is

(2) $$\begin{array}{c} E_{W}=E_{I}. \end{array}$$

Work of external forces (*E*_*W*_) is defined by the following formula:

(3) EW=12∫0a∫0b[Nx(∂w∂x)2+Nx(∂w∂x)2+2Nxy∂w∂x∂w∂y]dx dy   −∫0a∫0bwqdx dy.

The internal deformation energy (*E*_*I*_) is

(4) EI=D2∫0a∫0b{(∂2w∂x2+∂2w∂y2)2 −2(1−υ)[∂2w∂x2∂2w∂y2−(∂2w∂x∂y)2]}dx dy.

A necessary and sufficient condition for plate in order to be in stable equilibrium position is that the total energy *E* has a minimum. Total energy of plate whose components are given by (3) and (4) and in pursuance of [1, 2] is

(5) E=EW+EI=12∫0a∫0b[Nx(∂w∂x)2+Nx(∂w∂x)2+2Nxy∂w∂x∂w∂y]dx dy+D2∫0a∫0b{(∂2w∂x2+∂2w∂y2)2−2(1−υ)×[∂2w∂x2∂2w∂y2−(∂2w∂x∂y)2]}dx dy−∫0a∫0bwqdx dy.

First term refers to the deformation energy or to work performed by forces acting in the plane of the plate. The induction of these forces is a consequence of boundary conditions or restrictions of plate; their effect has passive character, and it manifests itself by reducing deflection, and thus reducing the deformation energy plate, too. An example is limited supporting plate, that is, plate supported on fixed supports to prevent extension caused by the deflection of plate. The occurrence of passive plane forces is always connected to the phenomenon of deformation or internal energy and it is characteristics of geometry, material, and boundary conditions of plate. Conversely, if the plate is exposed to external planar load then their effect is active because initiate strain and the corresponding energy is classified as external work.

### 3.2. Approximate Model of Planar State of Stress of Plate with Rectangular Hole

**Figure 1:**
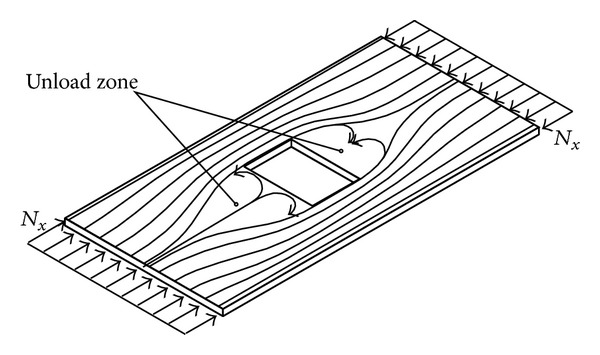
Redistribution of stress flow of plate with a hole.

To determine the work carried out by plane compression forces (3) it is necessary to know their distributions across the whole plate surface. If a plate is of constant thickness, with no holes, then the distribution of these forces is uniform and this case of load is the easiest case for studying stability. In case where there is a hole on the plate and/or stepwise variable thickness, disregarding the edges of plates exposed to constant planar load, there is a redistribution of these forces in the highly nonuniform functions. This phenomenon is explained by the existence of discontinuous changes through which the load cannot be transferred, but it is diverted to other areas of plates that are “bottlenecks” in the transfer of load. These are the most usually areas above and below the hole in whose immediate vicinity there is a concentration of the load, while in front of and back of the plate density of load stream lines decreases (Figure 1).

**Figure 2:**
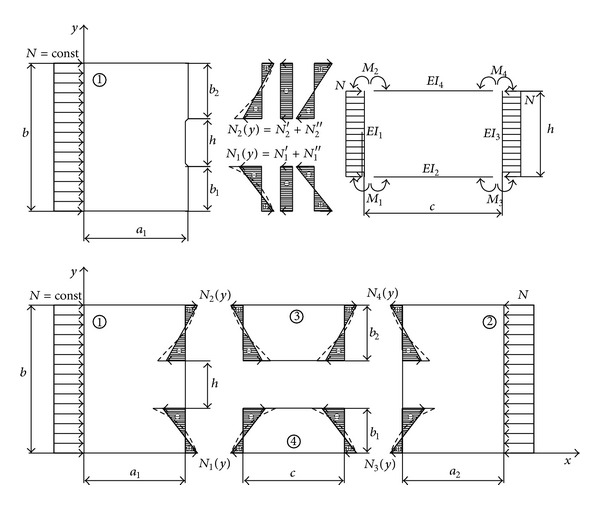
Model of planar state of stress of plate with rectangular hole.

Model of planar state of plate with rectangular hole (Figure 2) is formed under the following conditions:

- distribution of load at place where elements of plate are separated is linearized;
- the effect of the transversal forces along the connecting line among the elements of plate is ignored.

Justification for these assumptions is reflected in the fact that linearized force function *N*_*x*_ has the same mean value as real distribution that the difference of the external work is a small value in comparison to the work of real load. External shearing forces do not affect the plate and the induced deformation energy due to the fact that shear stress is small value in comparison to the normal stress. 

The components of the reaction force *N*_1_′(*y*) and *N*_2_′(*y*) defined by (6) and (7) are determined from the equilibrium condition of plate element “1” plates:

(6) N1′(y)=bb1b−b22b−b1−b2N=f1N, 0≤y≤b1

(7) N2′(y)=bb2b−b12b−b1−b2N=f2N, (b−b2)≤y≤b.

Elasticity of plate affects the induction of additional reactions of element “1” which manifest as moments (8) and linear variable force (9):

(8) $$\begin{array}{c} M_{1}=\frac{s_{2}e_{2}+s_{4}e_{1}}{s_{2}s_{3}-s_{1}s_{4}}N=g_{1}N,\\ M_{2}=\left(\frac{e_{1}}{s_{2}}+\frac{s_{1}}{s_{2}}\frac{s_{2}e_{2}+s_{4}e_{1}}{s_{2}s_{3}-s_{1}s_{4}}\right)N=g_{2}N,\\ M_{3}=\left[\frac{t_{1}}{r_{3}}-\frac{r_{2}}{r_{3}}\frac{e_{1}}{s_{2}}-\frac{s_{2}e_{2}+s_{4}e_{1}}{s_{2}s_{3}-s_{1}s_{4}}\left(\frac{s_{1}}{s_{2}}\frac{r_{2}}{r_{3}}+\frac{r_{1}}{r_{3}}\right)\right]N=g_{3}N,\\ M_{4}=\left[\frac{t_{1}}{r_{6}}-\frac{r_{8}}{r_{6}}\frac{e_{1}}{s_{2}}-\frac{s_{2}e_{2}+s_{4}e_{1}}{s_{2}s_{3}-s_{1}s_{4}}\left(\frac{s_{1}}{s_{2}}\frac{r_{8}}{r_{6}}+\frac{r_{2}}{r_{6}}\right)\right]N=g_{4}N. \end{array}$$

The coefficients *r*_1,…,8_, *s*_1,…,4_, *e*_1,…,2_, and *t*_1,…,2_ are defined in Appendix A.

Distribution of linear variable forces, caused by the influence of the moments of elastic clamp, is given by

(9) $$\begin{array}{c} N_{1}^{\prime \prime }\left(y\right)=\left(\frac{2y}{b_{1}}-1\right)\frac{2M_{1}}{b_{1}}=f_{1}N,\;0\leq x\leq b_{1},\\ N_{1}^{\prime \prime }\left(y\right)=\left(\frac{2\left(b-y\right)}{b_{2}}-1\right)\frac{2M_{2}}{b_{2}},\;\left(b-b_{2}\right)\leq x\leq b. \end{array}$$

The distribution of reaction forces (6), (7), and (9) along *x* = *a*_1_ is stepwise variable, which means that at part *b*_1_ < *y* < (*b*_1_ + *h*) does not act load. Developing into a single trigonometric series and by their adding coming up are to unique function (12) which determines the conditions at the edge *x* = *a*_1_ of segment “1” of plate is obtained:

(10) N1(x=a1,y) =∑n=1∞[4f1Nnπsinnπb12bsinnπb12b−8g1Nn2π2b12×cos⁡nπ2(nπcos⁡nπb12b−2bb1sinnπb12b)]×sinnπyb =N∑n=1∞Pnsinnπyb,

(11) N2(x=a1,y) =∑n=1∞[4f2Nnπsinnπ(2b−b2)2bsinnπb22b +8g2Nn2π2b22cos⁡nπ(2b−b2)2b ×(nπcos⁡nπb22b−2bb2sinnπb22b)]×sinnπyb=N∑n=1∞Qnsinnπyb,

(12) N(x=a1,y)=N1(x=a1,y)+N2(x=a1,y)=N∑n=1∞(Pn+Qn)sinnπyb.

Defining a planar state of stress of plate *Airy* stress function is applied. This function has the following form:

(13) $$\begin{array}{c} \frac{\partial^{4}U}{\partial x^{4}}+\frac{\partial^{4}U}{\partial x^{2}\partial y^{2}}+\frac{\partial^{4}U}{\partial y^{4}}=0. \end{array}$$

A function that satisfies (13) is presented by the trigonometric series:

(14) U(x,y)=∑n=1∞[Ansinh⁡nπxb+Bn(nπxb)sinh⁡nπxb    +Cncosh⁡nπxb+Dn(nπxb)cosh⁡nπxb]   ×sinnπyb.

Planar forces *N*_*x*_ and *N*_*xy*_ are defined through the component stresses *σ*_*x*_ and *σ*_*xy*_ formulated by

(15) Nx=σxδ=1δ∂2U∂y2=π2δb2∑n=1∞{n2[Ansinh⁡nπxb+Bn(2cosh⁡nπxb+nπxbsinh⁡nπxb)+Cncosh⁡nπxb+Dn(2sinh⁡nπxb+nπxbcosh⁡nπxb)]}×sinnπyb,Nxy=σxyδ=−1δ∂2U∂x∂y=−π2δb2∑n=1∞{n2[Ancosh⁡nπxb+Bn(sinh⁡nπxb+nπxbcosh⁡nπxb)+Cnsinh⁡nπxb+Dn(cosh⁡nπxb+nπxbsinh⁡nπxb)]}×cos⁡nπyb.

Integration constants *A*_*n*_, *B*_*n*_, *C*_*n*_, and *D*_*n*_ are determined from the boundary conditions as follows:

(16) (1) Nx=N=2Nπ∑n=1∞1n(1−cos⁡nπ)sinnπyb=const,Nxy=0, x=0,(2) Nx=N∑n=1∞(Pn+Qn)sinnπyb, Nxy=0, x=a1.

By substitution of (16) in (15) required coefficients defined by the coefficients (17) are obtained;

(17) $$\begin{array}{c} A_{n}=A_{n}^{\prime }N,\\ B_{n}=B_{n}^{\prime }N,\\ C_{n}=C_{n}^{\prime }N,\\ D_{n}=D_{n}^{\prime }N, \end{array}$$

where

(18) $$\begin{array}{c} \alpha_{n}=\frac{n\pi a_{1}}{b}. \end{array}$$

Coefficients *A*_*n*_′, *B*_*n*_′, *C*_*n*_′, and *D*_*n*_′ are given in Appendix B.

The coefficients given by (17)-(18) are derived for element “1,” and for applying them to element “2” is necessary to correct factor *α*_*n*_ by ratio *a*_1_/*a*_2_, and then its value is *α*_*n*_ = *nπa*_2_/*b*. If the hole is not centric along the *x*-axis, that is, if the center of hole is nearer to one of the sides *x* = 0 or *x* = *b*, then the elements of plates “1” and “2” are different, which is manifested through coefficient *α*_*n*_. Especially, if the hole is centric, then we have *α*_*n*_ = *nπa*/2*b*.

### 3.3. Determining the Buckling Coefficient and Critical Stress

Analysis of elastic buckling of perforated plate by energy method is presented on the example of simply supported plate of ideal planar geometry, and without effect of transversal load, noting that this does not reduce the generality of the methodology. Deflection is assumed by trigonometric series (7), which satisfies the boundary conditions of a simply supported plate:

(19) $$\begin{array}{c} w\left(x,y\right)=\sum_{m=1}^{\infty }\sum_{n=1}^{\infty }A_{mn}\sin\frac{m\pi x}{a}\sin\frac{n\pi y}{b}. \end{array}$$

The work carried out by uniaxial variable load (*N*_*x*_) affecting the plate with a rectangular hole is

(20) EW=12∫ANx(∂w∂x)2dA  =12∫0a1∫0bNx(∂w∂x)2dx dy+12∫a2a∫0bNx(∂w∂x)2dx dy+12∫a1a2∫0b1Nx(∂w∂x)2dx dy+12∫a1a2∫b2bNx(∂w∂x)2dx dy.

The total external work is determined by the components that correspond to elements of plates “1–4,” according to Figure 2:

(21) EW,1=12∫0a1∫0bNx(∂w∂x)2dx dy=12π4m2n2a2b2δAmn2×(AnImn+BnJmn+CnKmn+DnLmn)=12π4m2n2a2b2δAmn2×(An′Imn+Bn′Jmn+Cn′Kmn+Dn′Lmn)N=εmnAmn2N,EW,2=12∫a−a2a∫0bNx(∂w∂x)2dx dy=12π4m2n2a2b2δAmn2×(EnVmn+FnWmn+GnRmn+HnTmn)=12π4m2n2a2b2δAmn2×(En′Vmn+Fn′Wmn+Gn′Rmn+Hn′Tmn)N=ρmnAmn2N.

Integral forms given by coefficients *I*_*mn*_, *J*_*mn*_, *K*_*mn*_, and *L*_*mn*_ which correspond to element “1” or by coefficients *V*_*mn*_, *W*_*mn*_, *R*_*mn*_, and *T*_*mn*_ belonging to element “2” are formulated in Appendix C:

(22) EW,3=12∫a1a2∫b−b2bN1(y)(∂w∂x)2dx dy=14Amn2QnNm2bπna2(c+amπcos⁡2mπpasinmπca)×[13(cos⁡3nπ−cos⁡nπ(b−b2)b)−(cos⁡nπ−cos⁡nπ(b−b2)b)]=φmnAmn2N,EW,4=12∫a1a2∫0b1N2(y)(∂w∂x)2dx dy=14Amn2PnNm2bπna2(c+amπcos⁡2mπpasinmπca)×[13(cos⁡3nπb1b−1)−(cos⁡nπb1b−1)]=ωmnAmn2N.

Adequate internal or deformation energy of plate consisted of bending and torsion of the components is given by

(23) EI=D2∫A{(∂2w∂x2+∂2w∂y2)2−2(1−υ)[∂2w∂x2∂2w∂y2−(∂2w∂x∂y)2]}dA.

Flexion component of the internal energy is

(24) EI,f=18DAmn2π4(m4a4+n4b4+2υm2n2a2b2)×[ab−(c−amπcos⁡2mπpasinmπca)×(h−bnπcos⁡2nπqbsinnπhb)]=λmnAmn2.

Torsional component of the internal energy is:

(25) EI,t=14D(1−υ)Amn2π4m2n2a2b2×[ab−(c+amπcos⁡2mπpasinmπca)×(h+bnπcos⁡2nπqbsinnπhb)]=μmnAmn2.

The plate is in a state of stable equilibrium if the following condition is satisfied:

(26) $$\begin{array}{c} \frac{\partial E_{T}}{\partial A_{mn}}=0,\;\text{resp.}\;\frac{\partial \left(E_{I,f}+E_{I,t}+E_{W}\right)}{\partial A_{mn}}=0, \end{array}$$

where the total potential energy of the plate is given by

(27) $$\begin{array}{c} E_{T}=E_{I,f}+E_{I,t}+E_{W}. \end{array}$$

Substitution of (20), (24), and (25) in (26) leads to (28) which defines the critical stress of elastic buckling:

(28) $$\begin{array}{c} \sigma_{\text{cr}}=\frac{N_{\text{cr}}}{\delta }=\frac{\lambda_{mn}+\mu_{mn}}{\varepsilon_{mn}+\rho_{mn}+\varphi_{mn}+\omega_{mn}}. \end{array}$$

The critical buckling force can be written in compact form:

(29) $$\begin{array}{c} \sigma_{\text{cr}}=k\frac{\pi^{2}D}{b^{2}\delta }, \end{array}$$

where *k* is the coefficient of elastic buckling and it can be presented as

(30) khole=λmn+μmnεmn+ρmn+φmn+ωmn×b2δπ2D.

Ratio of buckling coefficient of uniform plate and plate with a hole is defined by sensitivity factor *t* which is a measure of plate sensitivity to discontinuous changes in geometry. This factor is proportional to the reciprocal value of the coefficient of elastic buckling *k*_hole_, and it is important because it represents the ability of plate with hole and a particular form and orientation, to keep its behavior in the domain of elastic stability:

(31) $$\begin{array}{c} t=\frac{k_{\text{unhole}}}{k_{\text{hole}}}=\frac{4}{k_{\text{hole}}}. \end{array}$$

**Figure 3:**
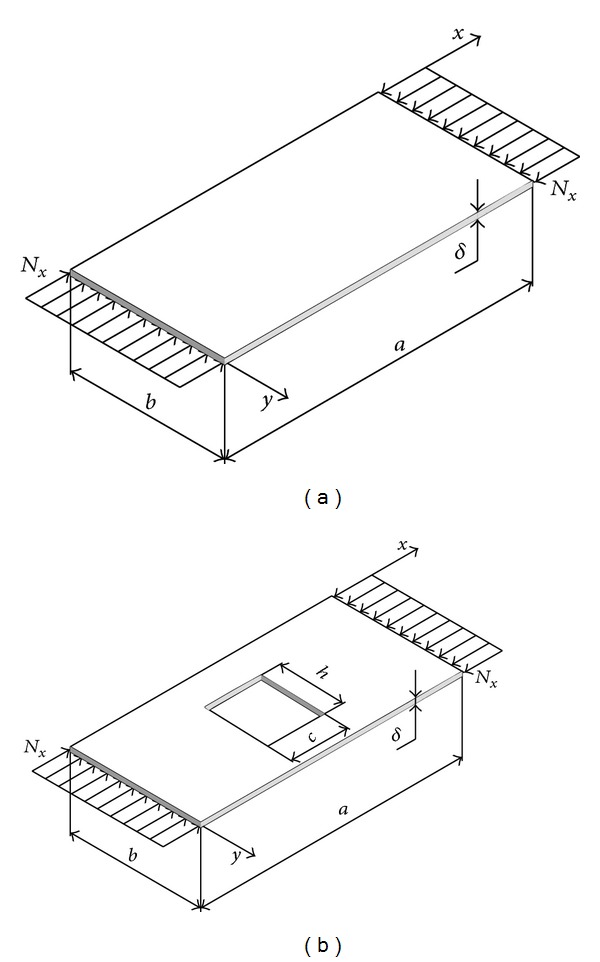
Uniaxial stressed rectangular plate: (a) uniform geometry; (b) with a rectangular hole.

Characteristic value of correction factor *t* is 1, corresponding to a uniform plate (Figure 3(a)). If the plate is with hole this value is adjusted by coefficient (1/*k*_hole_) and sensitivity factor *t* can be larger, smaller, or possibly equal to 1, depending on the values of influential parameters. 

## 4. Comparative Analysis and Verification of Results

By analyzing the mathematical model of elastic buckling it can be concluded that the work of external load has a crucial role in the stability of plate. This conclusion comes to the fore when the plate with elongated holes is considered. Specifically, then the change of deformation energy of plate is minor while the existence of the hole initiate redistribution of stresses and significant change in the external energy. The consequence of this phenomenon is the correction of buckling coefficient or sensitivity factor, reducing or increasing their value. In this paper, an analysis of the influential parameters for some of the characteristic forms will be considered.

### 4.1. The Case of Rectangular Hole

**Figure 4:**
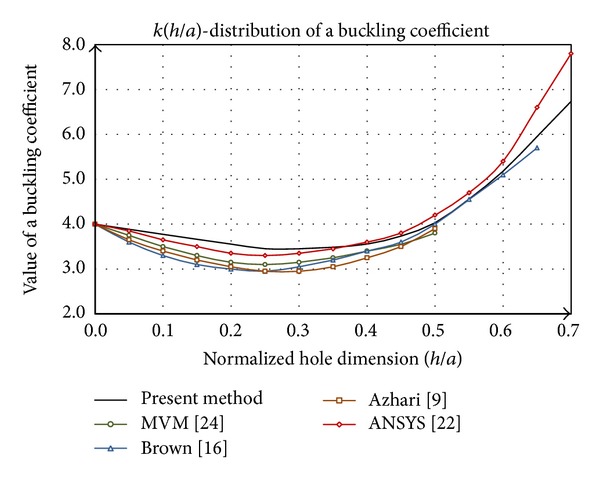
Comparative analysis of buckling coefficient of square plate with a rectangular hole.

Figure 4 shows the comparative analysis of the buckling coefficient by presented method with data for the square plate whose dimensions are *axa*, with centric rectangular hole dimensions of *cxh*, where *c* = 0,25*a* represents width of the hole. Commonly, the buckling coefficient *k* is expressed as a function of dimensionless value (*h*/*a*), where *h* is the height of the hole (Figure 3(b)). By analyzing the diagram (Figure 4) can be concluded that the data [9, 16, 22, 24] have the same distribution trend while the values differ.

The main difference between presented procedure and literature is reflected in the shape of the distribution until the values *y*/*a* = 0.25, where the function *k*(*h*/*a*) has a concave shape. According to [22] this form is linear, while other researches show that the function is convex in the whole domain. On the domain *h*/*a* > 0.25 function of buckling coefficient asymptotic tends to the distribution according to [16], while its values are the closest to the data corresponding to [22]. The research [18] by analyzing the vertical slotted hole, which is closest in shape to a rectangular hole, shows the existence of the concave side in the field to *h*/*a* = 0.25, as presented in the discussion on the slotted hole.

**Figure 5:**
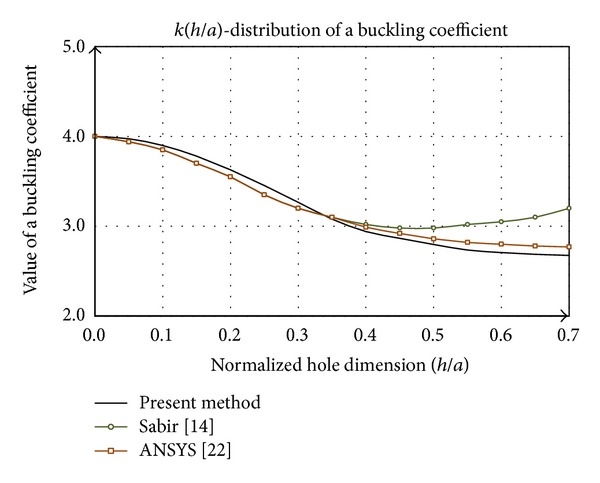
Comparative analysis of buckling coefficient of square plate with a square hole.

A special case of the previous considerations is shown in Figure 5, where the square plate with a centric square hole by dimensions of *dxd* is analyzed. Comparative analysis of this procedure is given in studies [14, 22]. By increasing dimensions of the hole in comparison with plate dimensions the buckling coefficient decreases permanently. The function *k*(*d*/*a*), the presented method, agrees well with the distribution corresponding to [22], both in terms of trend and quantity. According to [14] in the area above the *d*/*a* > 0.4 the buckling coefficient *k* first stagnates and then tends to grow slowly.

In the special case when there is no rectangular hole we have the case of geometric uniform simply supported and uniaxial stressed plate (Figure 3(a)) with buckling coefficient (32), corresponding to the data from [1, 2]:

(32) $$\begin{array}{c} k={\left(\frac{mb}{a}+\frac{a}{mb}\right)}^{2}. \end{array}$$

The minimum value of the critical stress is obtained for (*m*, *n*) = 1:

(33) $$\begin{array}{c} \sigma_{\text{cr}}\left(m,n=1\right)=4\frac{\pi^{2}D}{b^{2}}, \end{array}$$

where *k* = 4 is buckling coefficient of uniform square plate.

### 4.2. A Rectangular Plate with a Circular Hole

**Figure 6:**
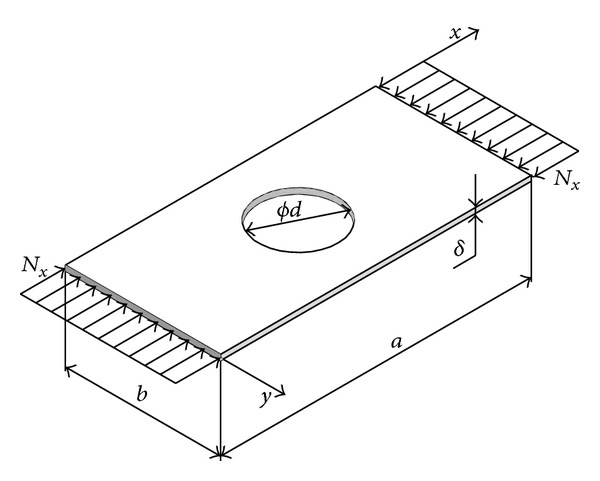
A rectangular plate with a circular hole.

Determination of the coefficient of elastic buckling of a rectangular plate with a circular hole (Figure 6) by previously presented procedure is not possible in a direct manner. The reason for this relates to the complexity of integrant due to circular contours and interdependent coordinates *x* and *y*. Therefore, it is necessary to use approximate solving approach that is based on the results of the previous analysis. Namely, the essence of this procedure is that the circular hole with diameter *d* is divided in *s* rectangular gaps with dimensions *h*_*i*_ and *c*_*i*_ whose positions are defined by *p*_*i*_ and *q*_*i*_. The number of rectangular gaps has to be large enough in order to follow the contour of circular hole in a best way. 

**Figure 7:**
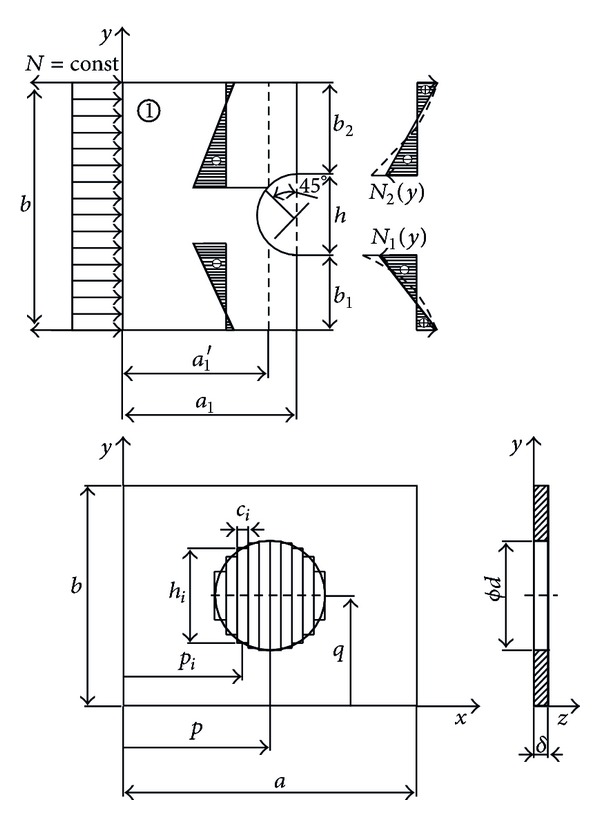
Distribution of the circular hole in rectangular gaps.

In order to be applicable for a circular hole, it is necessary to make a correction of model of planar state of stress developed for rectangular hole, according to Figure 7. Element “1” dimensions of *a*_1_ *xb* is adjusted to the length of *a*_1_′ to which the smaller influence of moments corresponds (distribution tends to be uniform form).

Defined geometric values of rectangular gaps (34) are used in the expression for the deformation energy (24) and (25) instead of values *p*, *c*, *q*, and *h*:

(34) $$\begin{array}{c} p_{i}=p-\frac{d}{2}+\frac{2i-1}{2}c,\;i=1,2,\ldots ,s,\\ c_{i}=\frac{d}{i},\;i=1,2,\ldots ,s,\\ q_{i}=q=\text{const},\\ h_{i}=\sqrt{d^{2}-{\left[\left(2i-1\right)c\right]}^{2}},\;i=1,2,\ldots ,s. \end{array}$$

**Figure 8:**
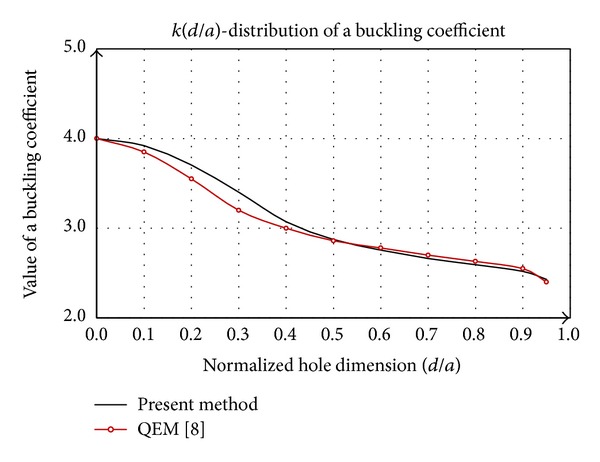
Comparative analysis of buckling coefficient of square plate with a circular hole.

The function of the buckling coefficient *k*(*d*/*a*) has a tendency to decline in value over the area of consideration (Figure 8). Progressive decline of value is in the interval *d*/*a* = (0.1–0.4) and values over 0.9. The obtained data are qualitatively and quantitatively consistent with [14], which justifies the assumptions in the process of model formation and its implementation to other similar contour shapes.

### 4.3. Rectangular Plate with a Slotted Hole

Research of plate with rectangular hole has shown that the orientation of the hole to the plate position has a great influence on the stability. On the other hand, analysis of plate with hole indicates an increase in work performed by an external force *N*_*x*_ causing a decrease of the buckling coefficient. Slotted hole represents a combination of the previous two cases.

**Figure 9:**
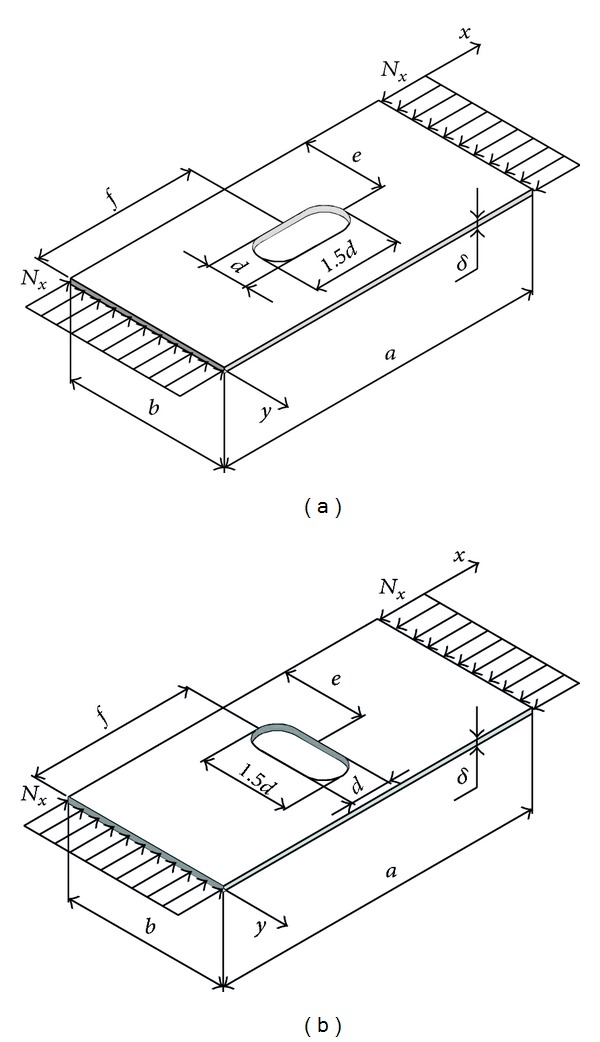
A rectangular plate with a slotted hole (a) horizontal (b) vertical orientation.

**Figure 10:**
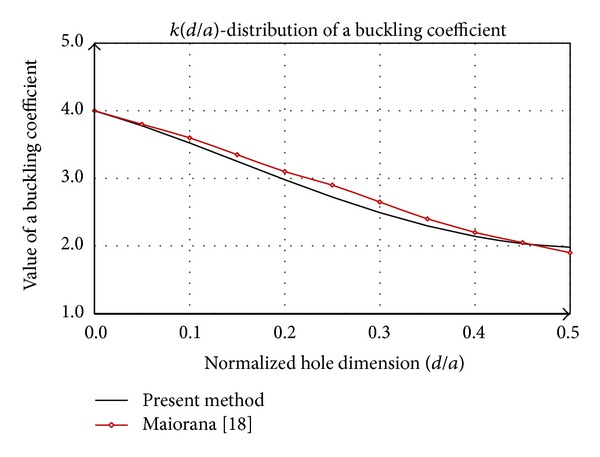
Comparative analysis of buckling coefficient of square plate with a slotted hole (horizontal position).

Analysis of buckling coefficient *k* indicates the dependence of the hole orientation.  Figure 9(a) corresponds to a horizontal hole position in relation to the direction of the force *N*_*x*_. Buckling coefficient according to [18], defined by FEM method, is linearly decreasing. The distribution of *k*(*d*/*a*) obtained by the presented methodology has the same trend as the maximum deviation of 5% for *d*/*a* = 0.25 (Figure 10). 

**Figure 11:**
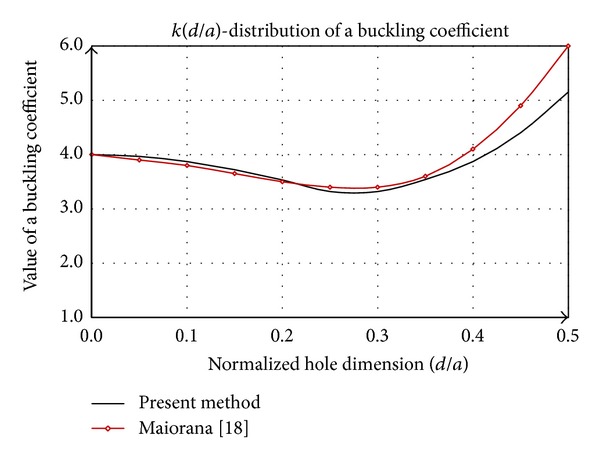
Comparative analysis of buckling coefficient of square plate with a slotted hole (vertical position).

However, if the hole is rotated by an angle of 90° (Figure 9(b)) while the other features unchanged, the behavior of the plate is very different. The function *k*(*d*/*a*) can be divided into two parts in the domain of examination. The first part refers to the interval [0,0.2], where *k* is a concave function and it decreases regressively with increasing *d*/*a*. In the domain [0.2,0.5] function has convex form that affects primarily the stagnation and then to progressively increase. Functions of buckling coefficients for the case with slotted hole and rectangular hole are analogous. The value of *k* is larger for the rectangular hole in relation to the slotted hole for the same value of dimensionless argument as a result of lower external work (Figure 11).

## 5. The Influence of the Hole on the External Work and Elastic Stability of Plate

Previously it was concluded that the work of external forces dominantly influence the elastic stability of plate. Deformation work of plate exclusively depends on the hole size, while the size, position, and orientation of the hole have secondary impact. Deformation work is a linear function of plate rigidity. Distributions of planar compressive forces cause induction of external work, so their identification is crucial in analysis of stability. Model of planar state of stress was developed for the rectangular hole; it was implemented with correction to circular and slotted form. The analysis carried out this section clearly shows that increasing of size of the one-dimensional hole (square and circular) leads to reduction in elastic stability. In the case of two-dimensional holes (rectangular shape and slotted) it should distinguish horizontal and vertical orientation. The first case is analogous to the previous analysis, while the second variant affects the elastic stability. It firstly decreases and then increases dominantly and even exceed the value of 4, which is characteristic for the plate without hole. Rectangular hole whose greater dimension is normal to the direction of load action is exposed to considerable stress concentration, which is negative in terms of static, but it is perfect condition for stability. Considering that the work of the external compression forces directly affects the buckling coefficient, it is necessary to introduce the coefficient of external work *λ*, which presents the absorbed energy caused by the shape of the hole in relation to the uniform plate. 

The coefficient of external work is defined as the ratio of external energy of plate with hole and uniform geometry, through relation

(35) $$\begin{array}{c} \lambda =\frac{E_{W,\text{hole}}}{E_{W,\text{unhole}}}. \end{array}$$

**Figure 12:**
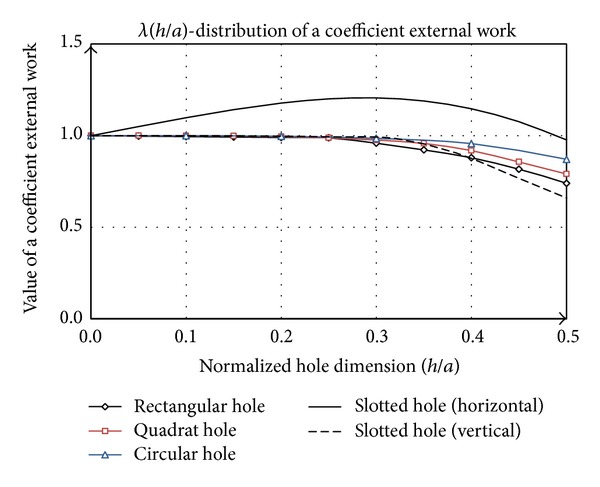
Change of coefficient *λ* in relation to the parameter (*h*/*a*) for the considered hole.

Value of coefficient *λ* for slotted hole in the horizontal position is greater than 1 in the interval from 0 to 0.5 (Figure 12). This value decreases for larger domains. Coefficient *λ* for the other shapes of hole has a tendency to fall, since their shape affects the reduction of work of compressive forces in relation to the uniform plate. The minimum value of *λ* is for the vertical orientation of slotted hole.

**Figure 13:**
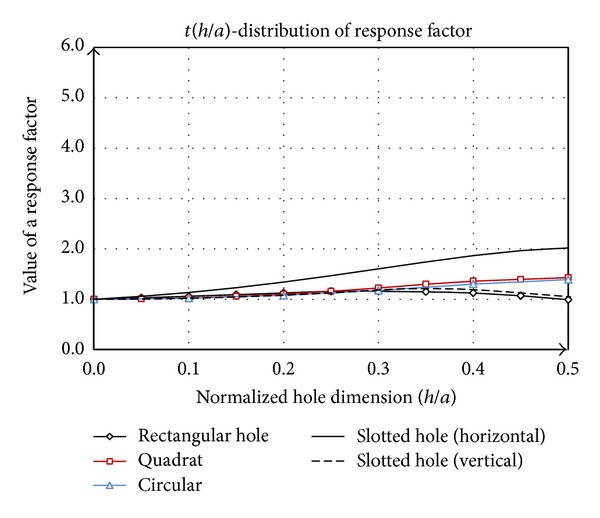
Sensitivity factor *t* as a function of the (*h*/*a*) for the holes of the considered forms.

There is certain similarity between the diagrams shown in Figures 12 and 13. Slotted hole in horizontal position has the most sensitivity. This form redistributes the load above and below the hole over nominal value of *N*, while in front of and behind the hole circular configuration prevents turbulence of fluid flow and distribution near the value *N*_*x*_ is formed. Because the value of *E*_*w*_ is greater than the value corresponding to the uniform plate, where the pressure distribution along the plate corresponds to the nominal value of *N*_*x*_. The situation of plate with rectangular hole is opposite to the above mentioned. As a result, we have maximum sensitivity for horizontal orientation slotted shape, while the same shape rotated by 90° has a minimum. Other variations are between these two cases.

The diagram given in Figure 13 is a basic guideline for selection of the hole shape in terms of the plate stability. Vertical position of slotted hole (Figure 9(b)), in this respect represent of optimal shape, which is important in the construction process of thin-walled supporting structures.

## 6. Conclusions

In this paper the problem of elastic stability of plate with a hole using a mathematical model developed on the basis of the energy method has been addressed. The analysis includes consideration of four types of holes: rectangular, square, circular, and slotted (the combination of rectangular and circular). The existence of the hole reduces the deformation energy of plate, depending on the form which most commonly affects the decrease in external work due to compressive forces. The dominant factor on the buckling coefficient *k* is the work of external forces. That is the reason why coefficient *λ* which manifests absorption ability of plate with the hole in relation to the uniform plate is defined. Through the exposed methodology analyzed mechanism of elastic stability of plates using energy balance in a state of stable equilibrium is analyzed. The mathematical identification of influential parameters on stability which include dimensions and type of plate material, shape, size, position, and orientation of the hole was carried out. 

Verification of results of the presented method has been verified by studies [9, 14, 16, 18, 22, 24]. Sensitivity factor *t* defines criteria for selection of the best form of hole in terms of stability. Results of the application of this study are important for the optimization of the structures, with special emphasis on thin-walled erforated elements of high bay warehouses in order to increase the economy of distribution of supply chains. The developed model (Section 3) is applicable to the methodology [31] and it can be implemented in order to further studying of the phenomenon of buckling of thin-walled structures.

## Appendices

### A.

Consider

(A.1) $$\begin{array}{c} s_{1}=\frac{r_{2}r_{7}}{r_{6}}+\frac{r_{1}r_{5}}{r_{3}},\;\;s_{2}=r_{6}-\frac{r_{7}r_{8}}{r_{6}}-\frac{r_{2}r_{5}}{r_{3}},\\ s_{3}=r_{3}-\frac{r_{2}r_{5}}{r_{6}}-\frac{r_{1}r_{4}}{r_{3}},\;\;s_{4}=\frac{r_{5}r_{8}}{r_{6}}+\frac{r_{2}r_{4}}{r_{3}},\\ e_{1}=t_{2}-\left(\frac{r_{5}}{r_{3}}+\frac{r_{7}}{r_{6}}\right)t_{1},\;\;e_{2}=t_{2}-\left(\frac{r_{5}}{r_{6}}+\frac{r_{4}}{r_{3}}\right)t_{1},\\ r_{1}=\frac{c}{3EI_{2}}+\frac{h}{3EI_{1}},\;\;r_{2}=\frac{h}{6EI_{1}},\\ r_{3}=\frac{c}{6EI_{2}},\;\;r_{4}=\frac{c}{3EI_{2}}+\frac{h}{3EI_{3}},\\ r_{5}=\frac{h}{6EI_{3}},\;\;r_{6}=\frac{c}{6EI_{4}},\\ r_{7}=\frac{c}{3EI_{4}}+\frac{h}{3EI_{3}},\;\;r_{8}=\frac{c}{3EI_{4}}+\frac{h}{3EI_{1}},\\ t_{1}=\frac{h^{3}}{24EI_{1}},\;\;t_{2}=\frac{h^{3}}{24EI_{3}},\\ I_{1}=\frac{\delta a_{1}^{3}}{12},\;\;I_{2}=\frac{\delta b_{1}^{3}}{12},\\ I_{3}=\frac{\delta a_{2}^{3}}{12},\;\;I_{4}=\frac{\delta b_{2}^{3}}{12}. \end{array}$$

### B.

Consider

(B.1) An′=[αnsinh⁡2αn+sinh⁡αn+αncosh⁡αnsinh⁡2αn−αn2×2nπ(1−cos⁡nπ)−sinh⁡αn+αncosh⁡αnsinh⁡2αn−αn2Pn+Qnn2]δb2π2,Bn′=[sinh⁡2αn−αnsinh⁡αn(cosh⁡αn+1)sinh⁡2αn−αn2×2nπ(1−cos⁡nπ)+αnsinh⁡αnsinh⁡2αn−αn2×Pn+Qnn2]δb2π2,Cn′=[2αnsinh⁡αn(cosh⁡αn+1)−sinh⁡2αn−αn2sinh⁡2αn−αn2×2nπ(1−cos⁡nπ)−2αnsinh⁡αnsinh⁡2αn−αn2×Pn+Qnn2]δb2π2,Dn′=[−αnsinh⁡2αn+sinh⁡αn+αncosh⁡αnsinh⁡2αn−αn2×2nπ(1−cos⁡nπ)+sinh⁡αn+αncosh⁡αnsinh⁡2αn−αn2×Pn+Qnn2]δb2π2.

### C.

Consider

(C.1)

## References

[1] Timoshenko SP, Woinowsky-Krieger S (1959). *Theory of Plates and Shells*.

[2] Timoshenko SP, Gere JM (1961). *Theory of Elastic Stability*.

[3] Allen HG, Bulson PS (1980). *Background To Buckling*.

[4] Gerstle KH (1967). *Basic Structural Design*.

[5] Radosavljević V, Dražić M (2010). Exact solution for buckling of FCFC stepped rectangular plates. *Applied Mathematical Modelling*.

[6] John Wilson A, Rajasekaran S (2012). Elastic stability of all edges simply supported, stepped and stiffened rectangular plate under uniaxial loading. *Applied Mathematical Modelling*.

[7] Paik JK, Thayamballi AK (2000). Buckling strength of steel plating with elastically restrained edges. *Thin-Walled Structures*.

[8] Zhong H, Pan C, Yu H (2011). Buckling analysis of shear deformable plates using the quadrature element method. *Applied Mathematical Modelling*.

[9] Azhari M, Shahidi AR, Saadatpour MM (2005). Local and post local buckling of stepped and perforated thin plates. *Applied Mathematical Modelling*.

[10] Petrišič J, Kosel F, Bremec B (2006). Buckling of plates with strengthenings. *Thin-Walled Structures*.

[11] Bedair OK, Sherbourne AN (1995). Plate/stiffener assemblies under nonuniform edge compression. *Journal of Structural Engineering*.

[12] Mizusawa T, Kajita T, Naruoka M (1980). Vibration and buckling analysis of plates of abruptly varying stiffness. *Computers and Structures*.

[13] Singh JP, Dey SS (1990). Variational finite difference approach to buckling of plates of variable stiffness. *Computers and Structures*.

[14] Sabir AB, Chow FY, Morris LJ Elastic buckling of flat panels containing circular and square holes.

[15] Brown CJ, Yettram AL (1986). The elastic stability of square perforated plates under combinations of bending, shear and direct load. *Thin-Walled Structures*.

[16] Brown CJ, Yettram AL, Burnett M (1987). Stability of plates with rectangular holes. *Journal of Structural Engineering*.

[17] Brown CJ (1990). Elastic buckling of perforated plates subjected to concentrated loads. *Computers and Structures*.

[18] Maiorana E, Pellegrino C, Modena C (2009). Elastic stability of plates with circular and rectangular holes subjected to axial compression and bending moment. *Thin-Walled Structures*.

[19] Harik IE, Andrade MG (1989). Stability of plates with step variation in thickness. *Computers and Structures*.

[20] Bui HC (2009). Buckling analysis of thin-walled sections under general loading conditions. *Thin-Walled Structures*.

[21] Brown CJ, Yettram AL (2000). Factors influencing the elastic stability of orthotropic plates containing a rectangular cut-out. *Journal of Strain Analysis for Engineering Design*.

[22] El-Sawy KM, Nazmy AS (2001). Effect of aspect ratio on the elastic buckling of uniaxially loaded plates with eccentric holes. *Thin-Walled Structures*.

[23] Moen CD, Schafer BW (2009). Elastic buckling of thin plates with holes in compression or bending. *Thin-Walled Structures*.

[24] Rahai AR, Alinia MM, Kazemi S (2008). Buckling analysis of stepped plates using modified buckling mode shapes. *Thin-Walled Structures*.

[25] Shanmugam NE, Lian VT, Thevendran V (2002). Finite element modelling of plate girders with web openings. *Thin-Walled Structures*.

[26] Bradford MA, Azhari M (1995). Buckling of plates with different end conditions using the finite strip method. *Computers and Structures*.

[27] Lau SCW, Hancock GJ (1986). Buckling of thin flat-walled structures by a spline finite strip method. *Thin-Walled Structures*.

[28] Cheung YK, Au FTK, Zheng DY (2000). Finite strip method for the free vibration and buckling analysis of plates with abrupt changes in thickness and complex support conditions. *Thin-Walled Structures*.

[29] Bisagni C, Vescovini R (2009). Analytical formulation for local buckling and post-buckling analysis of stiffened laminated panels. *Thin-Walled Structures*.

[30] Stamatelos DG, Labeas GN, Tserpes KI (2011). Analytical calculation of local buckling and post-buckling behavior of isotropic and orthotropic stiffened panels. *Thin-Walled Structures*.

[31] Djelosevic M, Gajic V, Petrovic D, Bizic M (2012). Identification of local stress parameters influencing the optimum design of box girders. *Engineering Structures*.

[32] Djelosevic M, Tanackov I, Kostelac M, Gajic V, Tepic J (2013). Modeling elastic stability of a pressed box girder flange. *Applied Mechanics and Materials*.

